# The Proportion of Anemia Associated with Iron Deficiency in Low, Medium, and High Human Development Index Countries: A Systematic Analysis of National Surveys

**DOI:** 10.3390/nu8110693

**Published:** 2016-11-02

**Authors:** Nicolai Petry, Ibironke Olofin, Richard F. Hurrell, Erick Boy, James P. Wirth, Mourad Moursi, Moira Donahue Angel, Fabian Rohner

**Affiliations:** 1 GroundWork, Fläsch 7306, Switzerland; nico@groundworkhealth.org (N.P.); ioo523@mail.harvard.edu (I.O.); james@groundworkhealth.org (J.P.W.); 2 Department of Epidemiology; Harvard School of Public Health, Boston, MA 02115, USA; 3 Laboratory of Human Nutrition, Institute of Food, Nutrition, and Health, ETH Zurich, Zurich 8092, Switzerland; richard.hurrell@hest.ethz.ch; 4 Harvest Plus, International Food Policy Research Institute, Washington, DC 20006, USA; E.Boy@cgiar.org (E.B.); m.moursi@cgiar.org (M.M.); m.angel@cgiar.org (M.D.A.)

**Keywords:** anemia, iron deficiency anemia, iron deficiency, determinants of anemia

## Abstract

Iron deficiency is commonly assumed to cause half of all cases of anemias, with hereditary blood disorders and infections such as hookworm and malaria being the other major causes. In countries ranked as low, medium, and high by the Human Development Index, we conducted a systematic review of nationally representative surveys that reported the prevalence of iron deficiency, iron deficiency anemia, and anemia among pre-school children and non-pregnant women of reproductive age. Using random effects meta-analyses techniques, data from 23 countries for pre-school children and non-pregnant women of reproductive age was pooled, and the proportion of anemia attributable to iron deficiency was estimated by region, inflammation exposure, anemia prevalence, and urban/rural setting. For pre-school children and non-pregnant women of reproductive age, the proportion of anemia associated with iron deficiency was 25.0% (95% CI: 18.0, 32.0) and 37.0% (95% CI: 28.0, 46.0), respectively. The proportion of anemia associated with iron deficiency was lower in countries where anemia prevalence was >40%, especially in rural populations (14% for pre-school children; 16% for non-pregnant women of reproductive age), and in countries with very high inflammation exposure (20% for pre-school children; 25% for non-pregnant women of reproductive age). Despite large heterogeneity, our analyses suggest that the proportion of anemia associated with iron deficiency is lower than the previously assumed 50% in countries with low, medium, or high Human Development Index ranking. Anemia-reduction strategies and programs should be based on an analysis of country-specific data, as iron deficiency may not always be the key determinant of anemia.

## 1. Introduction

Anemia is a public health problem that affects populations worldwide. To assess the global prevalence of anemia, the World Health Organization (WHO) established a global database containing population-based cross-sectional surveys and intervention studies. Using data collected from 1993 to 2005, it was estimated that about 1.6 billion people (a quarter of the world’s population) suffered from anemia, with the highest prevalence in pre-school children (PSC) and women of reproductive age (WRA) in Africa and South East Asia [1]. More recently, an analysis of survey data from 185 different countries (257 surveys in total) collected between 1990 and 2011 found that PSC and WRA still have the highest burden of anemia. Around 800 million PSC and WRA are anemic, and >60% of PSC in the African Region and >40% of WRA in the South East Asia Region had anemia [2].

The etiology of anemia is multifactorial and causes include nutritional deficiencies, chronic infections, inherited blood disorders, obesity, and chronic non-communicable diseases [3,4,5]. Dietary iron deficiency (ID), inherited blood disorders (sickle cell anemia, thalassemias), malaria, hookworm infestation, and schistosomiasis are the most frequent causes of anemia [6,7,8]. The contribution of these factors to the total anemia prevalence varies by population group, region, and general environmental settings [9]. As both ID and iron deficiency anemia (IDA) have serious health consequences, many countries have developed nutrition-specific and/or nutrition-sensitive strategies to increase individuals’ intake and absorption of iron [10]. Program planners need to know the proportion of total anemia that can potentially be addressed by improving iron status in order to estimate the impact and cost-effectiveness of a given intervention.

As representative data on iron status is unavailable for most countries, several approximations of the proportion of anemia due to ID have been made over the past several decades. Until recently, WHO had estimated that about half of the anemia burden of populations was caused by ID. Although the initial sources of this information are not easy to trace, it seems that the assumption was mainly based on a review conducted by DeMaeyer in 1985 [11]. DeMaeyer calculated the regional and global anemia prevalence for different population groups. Estimations of anemia due to ID were based on the assumption that anemia in male adults is not caused by ID. Subtracting the prevalence of anemia in male adults from the anemia prevalence in other population groups resulted in the fraction of anemia attributable to ID in the respective groups.

More recent meta-analyses on iron supplementation trials [2,12,13], examining the proportion of anemia resolved by iron supplementation, also found that about 50% of anemia was attributable to ID. Yet, the same publications also report that the proportion of the population with anemia attributable to ID changes with varying epidemiologic characteristics. For example, in regions where the burden of infectious diseases such as malaria, schistosomiasis, and HIV is high, and consequently the prevalence of anemia of infection (or inflammation) is high, the contribution of IDA to total anemia prevalence is comparably lower.

Therefore, for this work, we hypothesized that the proportion of the total anemia prevalence associated with ID is context-specific, and depends to a large extent on the exposure to inflammation, which in turn influences the magnitude of anemia prevalence. Using available nationally representative survey data in which ID, IDA, anemia, and inflammation prevalence were measured for PSC and WRA, we estimate the proportion of anemia associated with ID in countries ranked as low, medium, and high by the Human Development Index (HDI).

## 2. Materials and Methods

### 2.1. Search Strategy

We identified surveys that measured ID, IDA, and anemia prevalence amongst PSC and WRA by conducting a literature search in December 2015 in Web of Science, Google Scholar, and PubMed. Keywords used are as follows: iron AND ferritin (only in title); (anemia OR iron deficiency) AND (ferritin OR transferrin) AND (women OR children OR infants) AND survey; (anemia OR iron deficiency) AND (women OR children OR infants) AND survey. In order to identify studies not listed in the computerized databases, the reference lists of identified articles were also hand searched. Furthermore, we contacted the WHO, UNICEF, the Global Alliance for Improved Nutrition, and the Micronutrient Initiative to identify unpublished surveys. We also contacted authors of studies to obtain additional information on studies that were not presented in a suitable form but where indicators relevant to this analysis were assessed.

### 2.2. Inclusion and Exclusion Criteria

We included nationally representative surveys of PSC (6–59 months of age as the primary target age range, with some flexibility to include surveys with slightly older children; 10–75 months [14]) and WRA (15–49 years) in the analyses. Studies and surveys representative only of either urban or rural populations were also included to obtain separate estimates for urban and rural settings. To be included in the analysis, each report had to present the prevalence of anemia based on low hemoglobin levels as well as IDA (defined using low serum ferritin and low hemoglobin), where ideally, ferritin was adjusted for inflammation. The reported inflammation adjustment of included surveys was made by one of the following methods: (a) the approach proposed by Thurnham [15; 4-stage adjustment model: elevated C- reactive protein (CRP) only; both CRP and alpha-1-acid glycoprotein (AGP) elevated; or AGP elevated only]; (b) presenting IDA prevalence based on serum ferritin concentrations in a subsample without inflammation; (c) increasing the serum ferritin cut-off for ID to 20 μg/L for the whole study population (one survey [16]). In case IDA was not adjusted for inflammation by the authors, but the proportion of the population with elevated CRP or AGP was reported, IDA was adjusted applying an internal correction factor (see description below), and the survey was also included in the analyses.

We excluded studies that were not population-representative but used convenience or non-random sampling to select subjects, e.g., volunteers, clinic or hospital patients, and studies restricted to ethnic minorities, immigrant populations, or non-representative subgroups. We additionally excluded studies that did not report the sampling method used, studies whose sampling methodology could not be identified after all reasonable attempts, and studies that used sampling approaches that targeted specific hemoglobin levels, serum/plasma ferritin levels, other measures of body iron, anemia, iron deficiency, or iron deficiency anemia (e.g., studies that excluded severely anemic individuals, or were restricted to anemic individuals). Further, based on the very limited number of reports available, we excluded from analysis studies conducted in countries which were ranked as having a “very high Human Development Index” [17].

### 2.3. Estimating ID and IDA in Presence of Inflammation

We used the clinical case definitions as put forth by the authors of the different survey reports to define anemia, ID, IDA, and inflammation (% with elevated acute phase proteins (APPs)). In order to use data from seven surveys (out of 25) that reported IDA but did not correct for inflammation, we developed an algorithm to estimate ID and IDA in the presence of inflammation in these data sets. To correct the prevalence of IDA, we assumed that the proportion of ID in subjects who presented with no inflammation was the same as that in the subjects presenting with inflammation (elevated CRP or AGP as defined by the authors) and that the subjects with inflammation had not been diagnosed with IDA (i.e., anemia and serum ferritin < 15 μg/L in women; anemia and serum ferritin < 12 μg/L in children) because of inflammation-elevated ferritin levels. Thus, the IDA prevalence reported for the whole study population only reflected the prevalence in the subsample without inflammation. Based on these assumptions, we applied the same proportion of IDA to the segment of the population with inflammation as reported for the overall population, which resulted in an increase of IDA proportional to the % of the population with elevated APP:

$$\%IDA_{adjusted}=\%IDA_{unadjusted}+\frac{\left(\%IDA_{unadjusted}\cdot \%withelevatedAPP\right)}{100}$$

### 2.4. Grouping Countries by Inflammation Exposure

Since previous estimations indicate that the exposure to infection and chronic inflammatory conditions have an impact on the proportion of anemia associated with ID, and because the level of inflammation varies considerably between countries and population groups, we developed an inflammation-exposure index for children and women for each country using data obtained from external sources. These indices were constructed using factors known to contribute to inflammation. For children, the inflammation-exposure score was based on (a) prevalence of presumed and confirmed malaria cases [18,19]; (b) schistosomiasis prevalence [20]; and (c) an overall hygiene score based on the proportion of population using improved drinking water source and the proportion of the population using improved sanitation facilities (evenly weighted) as a proxy for the risk of enteric inflammation [21]. For women, the different factors used to estimate country-specific inflammation-exposure were (a) prevalence of presumed and confirmed malaria cases [18,19]; (b) HIV prevalence in adults [22]; (c) obesity prevalence in female adults [23]; (d) schistosomiasis prevalence [20]; and (e) an overall hygiene score based on the proportion of population using improved drinking water source and the proportion of the population using improved sanitation facilities (evenly weighted) as a proxy for the risk of enteric inflammation [21]. For 188 countries, information on the aforementioned factors was collected and combined into a single inflammation score. Using this score, countries were classified as having low, medium, high, or very high inflammation exposure (see Table S1 for a complete country list and references for the data used to calculate the index).

### 2.5. Data Synthesis

Data for estimating ID, IDA, and anemia prevalence were extracted from the available reports or publications into an Excel spreadsheet. We estimated the proportion of anemia associated with ID separately for PSC and WRA using the following calculation: (prevalence of IDA)/(prevalence of anemia). The country-specific proportions were then pooled using random effects meta-analysis of double arcsine transformed proportions (Freeman–Tukey transformation), which correctly estimates variances even in the presence of extreme proportions close to 0 or 1 and constrains confidence intervals to within the 0–1 range after back transformation [24,25]. Estimates were further pooled by region, inflammation exposure, setting (urban or rural), and the WHO classification of anemia burden for the country, separately for PSC and WRA. Analyses were conducted using R version 3.2.3 (The R Foundation, Vienna, Austria, 2015).

## 3. Results

### 3.1. Literature Research

In the first round, about 14,000 publications were identified according to the publication title and abstract. After screening the titles, abstracts, and the identification of surveys outside the literature search, 174 publications were selected for further investigation, from which 25 surveys met the inclusion criteria. In total, 23 national and 2 urban national representative publications reporting on anemia, IDA, and elevated CRP or AGP in PSC or WRA met our inclusion criteria. Surveys from Cambodia, Cameroon, and Afghanistan included 10.0%, 5.3%, and 17.5% pregnant women, respectively, as part of the WRA sample (0.8% of total study sample). All other surveys only included non-pregnant women of reproductive age in the analyses.

### 3.2. Country Inflammation Exposure Categorization

Based on the inflammation exposure index for children, globally, 21 countries were categorized into the very high exposure group, 28 countries into the high group, 33 countries in the medium group, and 106 countries in the low exposure group. For women, 20 countries were classified as being in the very high inflammation exposure category, 28 countries in the high inflammation exposure category, 58 countries in the medium exposure and 82 countries in the low exposure category. All countries in the very high exposure category for PSC and WRA are located in Sub-Saharan Africa. Similarly, the high exposure category is dominated by countries from Sub-Saharan Africa with 22 countries for PSC and 24 for WRA (Table S1).

### 3.3. Study Characteristics

Of the 25 included studies in the meta-analysis, 23 reported on children, and 23 on women. For PSC, seven surveys were conducted in Sub-Saharan Africa and South, East, and Southeast Asia, four in Latin America and the Caribbean, three in Central Asia, and two in the Middle East. Seven studies conducted in Sub-Saharan Africa, six in South, East, and Southeast Asia, four in Latin America and the Caribbean, four in Central Asia and two in Middle East reported on WRA. The studies included in our analysis were conducted between 2003 and 2014 (Table 1 and Table 2). The majority of countries included in the analysis fell into the low to medium inflammation-exposure index category, and only countries located in Sub-Saharan Africa were categorized into the very high exposure category.

### 3.4. Prevalence of Anemia, IDA, and ID among PSC

Anemia: As presented in Table 1, the total anemia prevalence in PSC ranged from 9.1% in Vietnam to over 76% in Sierra Leone, with a weighted mean prevalence of 34.9% (95% CI: 27.9, 42.0). The highest anemia prevalences were found in countries in the “very high” inflammation exposure category (i.e., Sierra Leone, Côte d’Ivoire, Mozambique, Liberia, and Cameroon). The estimated prevalence of anemia in countries with very high inflammation exposure was 66.0% (95% CI 59.6, 72.3), compared with 37.1% (95% CI: 29.1, 45.1), 25.7% (95% CI: 20.5, 30.9) and 24.4% (95% CI: 19.4, 29.3) in countries with high, medium, and low inflammation exposure, respectively. Anemia was most prevalent among PSC living in Sub-Saharan Africa (55.3%; 95% CI: 35.1, 75.6), whereas the lowest prevalence (22.9%; 95% CI: 19.9, 25.9) was found in Latin America. 

IDA: For IDA, the overall weighted mean prevalence was 9.6% (95% CI: 7.2, 12.0). The lowest prevalence was reported in Georgia (0.1%), followed by Vietnam (3.2%) and Mexico (3.4%). The highest prevalences were reported for Liberia (21.2%), the Philippines (18.7%), and Mozambique (17.3%). Similar to anemia prevalence, the highest prevalence of IDA was found in countries with very high inflammation exposure (15.5%; 95% CI: 10.5, 20.5), followed by high (14.2%; 95% CI: 10.3, 18.2), medium (8.4%; 95% CI: 6.8, 10.1), and low (5.1%; 95% CI: 2.5, 7.7) exposure to inflammation. The highest IDA prevalence was found in the Middle East (20.7%; 95% CI: 18.4, 23.0) and the lowest IDA prevalence in Central Asia (5.3%; 95% CI: 1.2, 9.5).

ID: The overall estimated prevalence of ID among PSC was 17.3% (95% CI: 13.8, 20.8). The lowest prevalence was reported in Georgia (0.2%), followed by Sierra Leone (5.2%) and Cambodia (7.6%), whereas the highest prevalences of ID were reported in children living in Nicaragua (37.9%), Liberia (29.8%) and the Philippines (27.9%). Prevalences of ID are comparable across the different inflammation exposure categories with about 20%, except for countries with low exposure to inflammation (11.0%; 95% CI: 3.4; 18.6). The highest prevalence of ID was found in South, East, and Southeast Asia (20.8%; 95% CI: 15.2, 26.4), the Middle East (20.7%; 95% CI: 18.4, 23.0), and Sub- Saharan Africa (20.5%; 95% CI: 15.4, 25.6) and lowest in Central Asia (8.9%; 95% CI: 1.9, 15.9).

### 3.5. Prevalence of Anemia, IDA, and ID among WRA

Anemia: The weighted mean anemia prevalence of all countries included in the analyses was 26.6% (95% CI: 22.2, 31.0), with the highest prevalences in Côte d’Ivoire (49.9%), Sierra Leone (44.8%), and Cambodia (42.7%), and the lowest anemia prevalence in Nicaragua (11.2%), Vietnam (11.6%), and Mexico (14.1%, Table 2). Similar to PSC, the prevalence of anemia among WRA was highest in countries with “very high” inflammation exposure (39.8%; 95% CI: 34.6, 45.0), and all countries were located in Sub-Saharan Africa. The mean anemia prevalence in the other inflammation exposure categories ranged from 21.9% to 27.8% and was lowest in countries with low inflammation exposure (21.9%; 95% CI: 16.5, 27.4). The prevalence of anemia among WRA was highest in Sub-Saharan Africa (35.9% (95% CI: 30.2, 41.6)) and lowest in Latin America and the Caribbean (17.2% (95% CI: 11.6, 22.7)).

IDA: The overall mean prevalence of IDA was 10.8% (95% CI: 8.0, 13.5). The pooled IDA prevalence was lowest (9.1%; 95% CI: 3.8, 14.4) in countries with “low” inflammation exposure, whereas ~12% of IDA was found in countries with medium (12.3%; 95% CI: 7.5, 17.2), high (12.3%; 95% CI: 11.0, 13.5), and very high (11.5%; 95% CI: 8.9, 14.1) exposure to inflammation. Highest prevalence of IDA was found in Central Asia (13.7% (95% CI: 3.4, 24.1)) and lowest in South, East, and Southeast Asia (7.8% (95% CI: 4.0, 11.7).

ID: The overall mean prevalence of ID was 20.8% (95% CI: 15.8, 25.7). The lowest ID prevalences were reported in countries with a very high burden of inflammation (17.8% (95% CI: 13.4, 22.3)) and highest in countries with medium exposure (25.2% (95% CI: 17.9, 32.5)), none of the latter being located in Sub-Saharan Africa. The prevalence of ID tended to be higher for women in the Middle East (33.8% (95% CI: 20.8, 46.9)) and Central Asia (26.1% (95% CI: 8.5, 43.7)) than for WRA in Sub-Saharan African (18.9% (95% CI: 15.6, 22.1)), Latin American and Caribbean (17.8% (95% CI: 8.6, 27.0)), and South, East, and South East Asian countries (17.3% (95% CI: 10.8, 23.9)).

### 3.6. Proportion of Anemia Associated with ID among PSC

**Figure 1:**
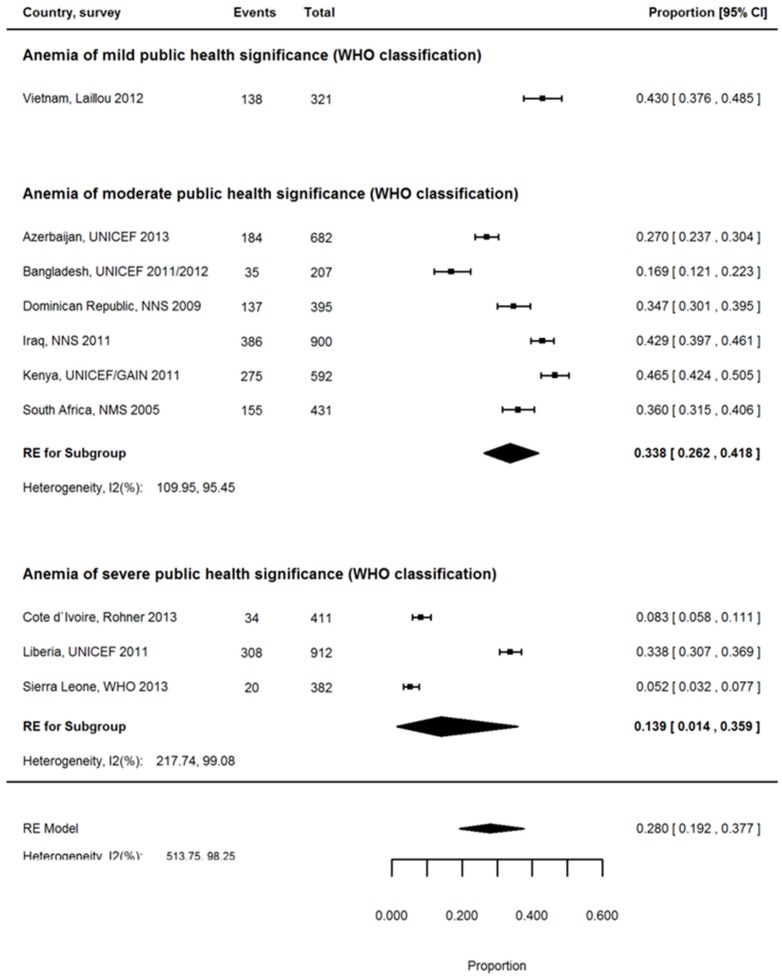
Proportion of anemia associated with ID (iron deficiency) in rural setting stratified by severity of anemia prevalence in PSC (pre-school children), pooled estimates.

The proportion of anemia associated with ID (Table 3) varied widely for PSC in the 21 countries with nationally representative data, and ranged from 0.2% for Georgia to 51% for Kenya with the pooled proportion for all countries being 24.6% (95% CI: 17.7, 32.2; Table 3, Figure S1). Regional estimates of the proportion of anemia associated with ID also varied considerably; 11.0% (95% CI: 0.2, 34.5) for Central Asia , 26.9% (95% CI: 18.9, 35.8) for Latin America and the Caribbean, 35.6% (95% CI: 20.2, 52.7) for the Middle East, 24.0% (95% CI: 17.6, 31.1) for South, East, and South-East Asia, and 28.1% (95% CI: 15.6, 42.6) for Sub-Saharan Africa (Figure S2). The proportion was slightly lower for children in rural settings than in urban settings (28.0% vs. 32.5%; Figure S3). Notably, the overall severity of anemia burden experienced by the country (estimated using the WHO classification) made little difference to the proportion of anemia associated with ID (25.7% for countries with moderate anemia burden and 21.7% for countries with severe anemia burden; Figure S4). However, after stratification for urban/rural setting, the proportion of anemia associated with ID decreased strongly with increasing anemia burden. In the rural setting, the proportion of anemia associated with ID decreased from 33.8% in countries where anemia is a moderate public health problem to 13.9% in countries where anemia is a severe public health problem. Similar results were found in the urban areas, decreasing from 39.8% to 26.0%, respectively (Figure 1). The inflammation exposure of the country did not change the proportion of anemia associated with ID (Figure S5).

### 3.7. Proportion of Anemia Associated with ID among WRA

**Figure 2:**
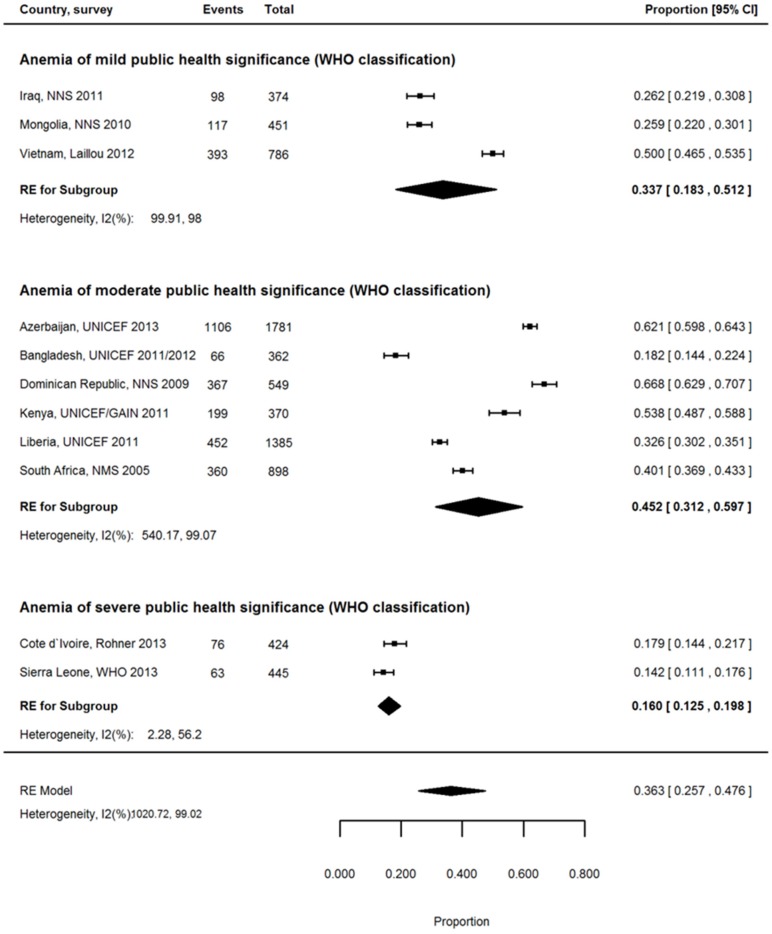
Proportion of anemia associated with ID in rural setting stratified by severity of anemia prevalence in WRA, pooled estimates.

The proportion of anemia associated with ID (Table 4) for WRA could be estimated in 22 countries with nationally representative surveys and was 36.7% overall (95% CI: 27.6, 46.3; Figure S6). Estimates tended to be lower for South, East, and South-East Asia (26.1%; 95% CI: 15.1, 38.8) and Sub-Saharan Africa 33.1% (95% CI: 22.5, 44.7) than for Latin America and the Caribbean (59.0%; 95% CI 54.3, 63.7; Figure S7). The proportion of anemia associated with ID was comparable for WRA in rural settings and urban settings (36.3% vs. 39.9%; Figure S8), yet the proportion of anemia associated with ID was considerably lower for countries with severe anemia burden (17.7%; 95% CI: 7.6, 30.9) than for countries with moderate (39.7%; 95% CI: 25.1, 55.3) and mild (44.7%; 95% CI: 33.9, 55.8) anemia burden (Figure S9), particularly in the rural setting (16.0%; 95% CI: 12.5, 19.8; Figure 2). The inflammation burden of the country did not significantly change the proportion, although countries with the highest inflammation exposure had the lowest average proportion (Figure S10).

## 4. Discussion

### 4.1. Anemia Attributable to ID

Our analyses—which focus on countries with a low, medium, and high HDI ranking—suggest that the overall proportion of anemia associated with ID is lower than commonly assumed. Consequently, our analyses suggest that the extent to which iron interventions alone can decrease anemia prevalence is also lower than previously believed. In particular, the relative impact of iron interventions may be smaller in populations with both high burden of anemia and very high inflammation exposure as the proportion of anemia associated with ID, tends to be relatively low, and iron absorption is restricted in the presence of inflammation. In such populations, more context-specific combinations of programmatic approaches to reduce the anemia may be needed. Our findings suggest that countries where anemia is a severe public health problem (i.e., anemia prevalence > 40%) should include infection prevention and control as a key strategy to reduce the prevalence of anemia. Iron interventions can and should be used in these contexts, but their implementation should be based on data from population representative surveys that help identify the target groups where ID is a public health problem.

To our knowledge, this is the first multi-country study to assess the proportion of anemia associated with ID by using a direct measure of ID (i.e., low ferritin concentration). Ferritin is currently the most widely used biomarker to assess the prevalence of ID. However, it is part of the acute phase response, and serum ferritin levels are increased in the presence of inflammation. If ferritin concentrations are not corrected for inflammation, the prevalence of ID can be underestimated by more than 14% [15]. In the present study, we exclusively included surveys measuring at least one further acute phase protein (e.g., CRP or AGP) and used adjusted ferritin concentrations for acute inflammation, as recommended by WHO [50].

Other research estimating the proportion of anemia amenable to iron interventions has been based on pooled reported shifts in hemoglobin concentrations after iron supplementation [2,10,12] or fortification [9] interventions. Using data from two meta-analyses on intermittent iron supplementation trials in children [51] and women [52], the WHO calculated a hemoglobin shift of 8 mg/L and 10.2 mg/L, respectively, resulting in global estimates of 42% for PSC and 49% for non-pregnant women of anemia attributable to ID. The hemoglobin shift estimates also included studies where participants received malaria and anthelminthic treatment in addition to iron, which might have resulted in an additional shift and an overestimation of anemia amenable to iron interventions. Furthermore, the included supplementation trials predominantly targeted specific hemoglobin levels (e.g., only anemic, only iron deficient anemic, or exclusion of severely anemic children) and would thus not be representative for entire populations. Also looking at the exclusive effect of iron supplementation, Bhutta and colleagues [10] found a decrease of anemia prevalence ranging from 38% to 62% in non-malarial regions and from 6% to 32% in malarial hyperendemic areas. A recent study of children aged 12–36 months of age conducted in a malaria endemic region in Côte d’Ivoire reported that iron-fortified porridge reduced the prevalence of iron deficiency, but had no effect on anemia prevalence, as malaria anemia seemed to override anemia associated with ID [53].

Using cause-specific shifts in hemoglobin after iron-fortification interventions, Kassebaum et al. [9] estimated that, in PSC, about 60% of anemia is associated with ID globally. To calculate the anemia attributable to ID, the authors compared the hemoglobin shift after iron fortification to the total shift of hemoglobin taking into account the prevalence of the different causes of anemia in 187 countries. The calculated hemoglobin shift of 6.05 mg/L was based on the results of a meta-analysis of seven iron-fortification comparisons drawn from five studies in India, Pakistan, South Africa, Guatemala, and Chile, none of those having a very high inflammation burden. Three of the comparisons were conducted in school-aged children, two of which had markedly higher Hb shifts (14 and 17 mg/L) than other studies included in the meta-analysis. These assumptions could explain some of the differences observed between our approach and the method by Kassebaum, particularly for PSC in countries with very high exposure to inflammation.

Though our analysis is not global and could not make estimations by WHO regions, some scoping comparisons between our findings and WHO’s estimates can be made. Globally, the WHO [2] estimates are markedly higher than our overall estimates of 25% and 37%, and this trend is consistent with our approximations of regional estimates. For children and non-pregnant women, WHO respectively estimated the proportion of anemia attributable to ID at 32% and 41% in the Africa Region and 41% and 54% in the South-East Asia Region [2]. Our estimates for Sub-Saharan African countries were 28% and 33% for PSC and WRA, and 24% and 26% for South-East Asia (see Figures S2 and S7). As countries in these two regions have a higher disease and inflammation risk, and sizable populations with hemoglobin disorders, our findings suggest that the Hb shift methodology employed by WHO could have overestimated the proportion of anemia associated with ID in these regions.

Understanding the contribution of ID to anemia is important to national-level programmers and public health professionals. In addition to helping design anemia-reduction strategies, the proportion of anemia attributable to ID is often used by health economists when estimating disease burden using disability-adjusted life years (DALYs) or similar metrics. As data on IDA is relatively scarce, DALY calculation methods [54] have recommended estimating the prevalence of IDA as 50% of the total anemia prevalence if national-level IDA data is not available. Our analysis suggests that, if available, national-level data should be used for estimates of disease burden. If national estimates are not available, a proportion of 25% and 37% can be temporarily used for PSC and WRA in countries with a low to high HDI until a survey measuring IDA prevalence can be conducted.

### 4.2. Heterogeneity of Estimates of ID Associated with Anemia

Our analyses also found that the proportion of anemia associated with ID is highly heterogeneous. Regional stratification could not explain the observed heterogeneity, which is likely owed to the large variability within regions. The most distinct variability was found in the region of Central Asia, with Georgia and Tajikistan exhibiting very low, and Uzbekistan and Azerbaijan very high proportions of anemia associated with ID. Reasons for those differences are unclear, since Azerbaijan and Georgia as well as Tajikistan and Uzbekistan are neighboring countries and have similar environmental settings, similar living standards, and comparable inflammation exposures. Thus, these differences remain to be elucidated by looking more closely at dietary habits as well as the etiology of anemia in those countries. Much less variability was found in Latin America and the Caribbean countries, allowing a more solid estimate for this region, with the proportion of anemia associated with ID ranging from 17% to 34% among PSC and 53% to 63% among WRA.

Similar to this, we found a comparably low heterogeneity in anemia associated with ID among children in South, East, and Southeast Asian countries (12%–35%), which was not the case for Sub-Saharan Africa. Further subgroup analyses that were stratified by burden of anemia showed that in countries with severe burden of anemia such as Sierra Leone, Côte d’Ivoire, and Liberia, the proportion of anemia associated with ID is significantly lower, especially in the rural populations (14% for PSC; 16% for WRA). This difference for rural populations might be explained by poor sanitary conditions that are often accompanied with increased prevalence of infection.

### 4.3. Limitations of Analysis Methodology

Our methodology has limitations that might have impacted the precision of our estimates. First, we were not able to use a consistent approach for correcting ferritin concentrations and ID/IDA prevalence for inflammation. The most frequently used correction factor, developed by Thurnham [15], is based on a meta-analysis looking at serum ferritin during different phases of the acute phase response (incubation, early convalescence, late convalescence) using CRP and AGP. The correction factor we developed for seven surveys showed a strong correlation with Thurnham’s correction factor, except when there is a high prevalence of elevated APP. In this case, our correction factor tends to be higher, resulting in a greater increase in ID and IDA prevalence. Some of the surveys included in our analysis only measured ID and IDA in subjects without inflammation. This substantially reduced the sample size of some surveys and thus might lead to biased results as their reported ID prevalence was not completely representative of the population. These different corrections may have under- or overestimated the prevalence of ID and IDA and thus the proportion of anemia associated with ID. Inflammation is a very complex process and it is perhaps simplistic to believe that one correction can fit all causes of inflammation. Currently, the Biomarkers Reflecting Inflammation and Nutritional Determinants of Anemia (BRINDA) Project is examining new approaches to adjust ferritin concentrations for inflammation. In addition to these efforts, greater research is needed to elucidate the relationship between inflammation and serum ferritin concentrations [55].

Second, our approach may have overestimated the proportion of anemia that can be resolved with iron interventions in malaria-endemic areas. To illustrate, anemia can often be multifactorial in the same person. Even in individuals where anemia is associated with ID, communicable diseases such as malarial infection can prevent anemia prevalence being decreased with iron interventions [53]. 

Lastly, our analyses contained sparse data from countries in the Eastern and Central Europe and the Pacific Regions, which had highest proportions of IDA according to the estimations made by Kassebaum et al [9]. As we could not identify suitable data from countries in these regions for our analysis, our overall estimate of the proportion of anemia associated with ID may be lower than global estimates.

## 5. Conclusions

Our analyses suggest that the all-encompassing estimate that 50% of anemia is attributable to ID is too high for PSC and WRA in countries with a low, medium, and high HDI ranking. Furthermore, we observed a large heterogeneity in the proportion of anemia associated with ID between countries in the same region. We therefore conclude that estimating the proportion of anemia attributable to ID using a fixed proportion (based on data from another country or hemoglobin shifts observed by supplementation studies) is inappropriate. Where possible, national surveys should measure ID and IDA prevalence so that program planners and national stakeholders can better understand the etiology of anemia in their country. Our assessment was limited to the 25 surveys that met our inclusion criteria. This relatively small number of surveys highlights the need to collect data on ID and IDA prevalence along with anemia on the national level. Ideally, this should be complemented by the assessment of other causes of anemia. Until a nationally representative survey can be conducted, public health and nutrition stakeholders can cautiously estimate that 25% and 37% of anemia is associated with ID in PSC and WRA, respectively. We argue that the current habit of assuming 50% of anemia attributable to ID should no longer be used for countries with a low to high HDI.

## Figures and Tables

**Table 1:** Anemia, iron deficiency (ID), and iron deficiency anemia (IDA) prevalence, and percentages of total anemia associated with ID, in pre-school children (PSC) in countries with nationally representative data, by country and year of survey completion.

Country	Region ^1^	Inflammation Exposure	Anemia		IDA		ID		Proportion of Anemia Associated with ID	Reference
			*n*	%	*n*	%	*n*	%	%	
Cameroon, 2012 ^2^	SSA	Very high	859	57.6	798	16.5	838	20.6	28.7	[26]
Côte d’Ivoire, 2007 ^2^	SSA	Very high	879	71.8	783	12.0	781	15.5	16.7	[27]
Kenya, 2011 ^2^	SSA	High	827	26.3	827	13.3	918	21.8	50.6	[28]
Liberia, 2011 ^2^	SSA	Very high	1445	59.1	1415	21.2	1416	29.8	35.9	[29]
Mozambique, 2012/2013 ^2,4^	SSA	Very high	962	70.8	917	17.3	917	19.3	24.4	[30]
Sierra Leone, 2013 ^2^	SSA	Very high	710	76.3	668	3.8	654	5.2	5.0	[31]
South Africa, 2005 ^3^	SSA	High	1049	28.9	768	12.3	821	21.4	42.6	[32]
Afghanistan, 2013 ^2^	SEA	Medium	728	44.9	728	13.7	728	26.1	30.5	[33]
Bangladesh, 2011/2012 ^2^	SEA	Medium	607	33.1	449	7.2	468	10.7	21.8	[34]
Cambodia, 2014 ^2^	SEA	High	485	51.7	485	6.0	485	7.6	11.6	[35]
Laos, 2006 ^2^	SEA	High	495	40.9	483	10.8	483	18.4	26.4	[36]
Mongolia, 2010 ^5^	SEA	Medium	433	23.9	433	5.0	433	21.4	20.9	[37]
Philippines, 2010 ^2^	SEA	High	1784	41.8	1784	18.7	1784	27.9	44.7	[38]
Vietnam, 2009 ^2^	SEA	Low	583	9.1	564	3.2	568	12.9	35.2	[14]
Dominican Rep., 2009 ^5^	LAC	Low	772	28.1	321	8.7	321	27.7	31.0	[39]
Ecuador, 2012 ^3^	LAC	Low	2046	25.7	1913	7.0	2045	10.6	27.2	[40]
Mexico, 2012 ^2^	LAC	Low	2352	20.4	2319	3.4	2319	13.9	16.7	[41]
Nicaragua, 2003–2005 ^5^	LAC	Medium	1420	20.1	731	6.9	731	37.9	34.3	[42,43]
Iraq, 2011 ^3^	ME	Medium	2275	21.6	2087	9.5	2087	20.2	44.0	[44]
Oman, 2005 ^3^	ME	Low	247	41.5	178	11.2	183	26.3	27.0	[45]
Azerbaijan, 2013 ^2^	CA	Medium	111	24.2	1460	6.5	1233	15.0	26.9	[46]
Georgia, 2010 ^5^	CA	Low	2222	23.9	1648	0.1	1648	0.2	0.3	[47]
Tajikistan, 2009 ^3^	CA	Low	2136	28.7	2136	8.6	2136	12.1	16.4	[48]

^1^ SSA: Sub-Saharan Africa South; SEA: South, East, and Southeast Asia; LAC: Latin America and the Caribbean; ME: Middle East; CA: Central Asia; ^2^ ID and IDA prevalence corrected by Thurnham [15]; ^3^ In-house correction factor based on reported prevalence of elevated CRP; ^4^ Urban/peri-urban setting only; ^5^ Reported for subsample without infection.

**Table 2:** Anemia, ID, IDA prevalence, and percentages of total anemia associated with ID, among women of reproductive age (WRA) in countries with nationally representative data, by country, and year of survey completion.

Country	Region ^1^	Inflammation Exposure	Anemia		IDA		ID		Proportion of Anemia Associated with ID %	Reference
			*n*	%	*n*	%	*n*	%		
Cameroon, 2012 ^2,3^	SSA	Very high	888	38.8	857	11.1	872	15.3	28.6	[26]
Côte d’Ivoire, 2007 ^2^	SSA	Very high	928	49.9	905	11.6	910	16.7	23.3	[27]
Kenya, 2011 ^2^	SSA	High	592	21.9	592	14.0	633	21.3	63.9	[28]
Liberia, 2011 ^2^	SSA	Very High	1955	33.2	1911	11.3	1911	19.6	34.0	[29]
Mozambique, 2012/2013 ^2,4^	SSA	Very high	1086	39.8	1068	16.1	1068	25.1	40.5	[30]
Sierra Leone, 2013 ^2^	SSA	Very high	871	44.8	827	6.1	774	8.3	13.6	[31]
South Africa, 2005 ^5^	SSA	High	2126	29.4	2126	11.8	1906	20.9	40.1	[32]
Afghanistan, 2013 ^2,3^	SEA	Medium	1187	40.4	1187	13.8	1187	24.0	34.2	[33]
Bangladesh, 2011/2012 ^2^	SEA	Low	1031	26.0	868	4.8	882	7.1	18.5	[34]
Cambodia, 2014 ^2,3^	SEA	Medium	450	42.7	450	2.2	450	2.9	5.2	[35]
Laos, 2006 ^2^	SEA	Medium	825	36.2	818	14.6	818	23.2	40.3	[36]
Mongolia, 2010 ^6^	SEA	Medium	892	14.4	767	3.0	767	28.2	20.8	[37]
Vietnam, 2009 ^2^	SEA	Low	1526	11.6	1522	5.4	1523	13.7	46.6	[14]
Dominican Rep., 2009 ^6^	LAC	Medium	1129	33.8	534	20.9	541	49.4	61.8	[39]
Ecuador, 2012 ^3^	LAC	Low	6958	16.9	6548	9.0	6957	15.5	53.3	[40]
Mexico, 2006 ^7^	LAC	Medium	2521	14.1	2521	8.1	2521	9.5	59.7	[16]
Nicaragua, 2003–2005 ^5,8^	LAC	Medium	1500	11.2	1500	6.9	1500	30.9	61.6	[42]
Iraq, 2011 ^5^	ME	Medium	1206	19.9	1152	5.7	1152	28.7	28.6	[44]
Oman, 2005 ^5^	ME	Low	352	38.8	338	29.0	342	51.1	74.7	[45]
Azerbaijan, 2013 ^2^	CA	Medium	2706	38.2	2621	23.8	2727	34.1	62.3	[46]
Georgia, 2010 ^6^	CA	Low	1721	24.1	1721	0.7	1721	1.6	2.9	[47]
Tajikistan, 2009 ^5^	CA	Low	2138	24.2	2138	2.2	2138	9.7	9.1	[48]
Uzbekistan, 2008 ^2^	CA	Low	2582	34.4	2582	21.8	2582	47.5	63.2	[49]

^1^ SSA: Sub-Saharan Africa South; SEA: South, East, and Southeast Asia; LAC: Latin America and the Caribbean; ME: Middle East; CA: Central Asia; ^2^ ID and IDA prevalence corrected by Thurnham [15]; ^3^ Women of reproductive age including pregnant women: Cameroon, 10.0%; Afghanistan, 17.5%, Cambodia, 5.3%; ^4^ Urban/peri-urban; ^5^ In-house correction factor based on reported prevalence of elevated CRP; ^6^ Reported for subsample without infection; ^7^ Cut-off for ID: sf ≤ 20μg/L; ^8^ Number of households surveyed.

**Table 3:** Proportion of anemia associated with ID among PSC stratified by region, anemia burden, inflammation exposure, and rural/urban setting, pooled estimates.

Stratification	%	(95% CI)	*I*^2^ (%)
Overall	24.6	(17.7; 32.2)	99.4
Regions ^1^			
SSA	28.1	(15.6; 42.6)	99.3
SEA	24.0	(17.6; 31.1)	95.4
LAC	26.9	(18.9; 35.8)	98.3
ME	35.6	(20.2; 52.7)	96.4
CA	11.0	(0.2; 34.5)	99.8
Anemia burden, all			
Severe (≥40%)	21.7	(13.8; 30.7)	98.4
Moderate (20%–39.9%)	25.7	(15.8; 37.2)	99.6
Anemia burden, rural			
Severe (≥40%)	13.9	(1.4; 35.9)	99.1
Moderate (20%–39.9%)	33.8	(26.2; 41.8)	95.5
Anemia burden, urban			
Severe (≥40%)	26.0	(13.7; 40.7)	98.9
Moderate (20%–39.9%)	39.8	(31.9; 48.0)	95.1
Inflammation exposure			
Very high	20.0	(8.0; 35.6)	99.2
High	31.7	(16.4; 49.4)	99.2
Moderate	29.6	(22.5; 37.2)	98.2
Low	19.6	(8.8; 33.4)	99.7
Setting			
rural	28.0	(19.2; 37.7)	98.3
urban	32.5	(25.1; 40.4)	98.0

^1^ SSA: Sub-Saharan Africa South; SEA: South, East, and Southeast Asia; LAC: Latin America and the Caribbean; ME: Middle East; CA: Central Asia.

**Table 4:** Proportion of anemia associated with ID among women of reproductive age stratified by region, anemia burden, inflammation exposure, and rural/urban setting, pooled estimates.

Stratification	%	(95% CI)	*I*^2^ (%)
**Women of reproductive age**			
Overall	36.7	(27.6; 46.3)	99.7
Regions ^1^			
SSA	33.1	(22.5; 44.7)	99.0
SEA	26.1	(15.1; 38.8)	99.1
LAC	59.0	(54.3; 63.7)	95.7
ME	51.8	(10.7; 91.4)	99.6
CA	29.7	(3.8; 66.7)	99.9
Anemia burden, all			
Severe (≥40%)	17.7	(7.6; 30.9)	98.8
Moderate (20%–39.9%)	39.7	(25.1; 55.3)	99.8
Mild (5%–19.9%)	44.7	(33.9; 55.8)	99.4
Anemia burden, rural			
Severe (≥40%)	16.0	(12.5; 19.8)	99.0
Moderate (20%–39.9%)	45.2	(31.2; 59.7)	99.1
Mild (5%–19.9%)	33.7	(18.3; 51.2)	98.0
Anemia burden, urban			
Severe (≥40%)	33.5	(8.3; 65.4)	99.5
Moderate (20%–39.9%)	37.4	(28.5; 46.8)	97.6
Mild (5%–19.9%)	52.0	(18.8; 84.2)	99.4
Inflammation exposure			
Very high	24.5	(16.1; 34.0)	98.0
High	52.0	(29.1; 74.4)	99.1
Moderate	40.3	(28.0; 53.2)	99.5
Low	35.3	(15.9; 57.6)	99.9
Setting			
rural	36.3	(25.7; 47.6)	99.0
urban	39.9	(30.2; 50.1)	98.8

^1^ SSA: Sub-Saharan Africa South; SEA: South, East, and Southeast Asia; LAC: Latin America and the Caribbean; ME: Middle East; CA: Central Asia.

## Supplementary Materials

The following are available online at http://www.mdpi.com/2072-6643/8/11/693/s1, Figure S1: Overall proportion of anemia associated with iron deficiency (ID) among pre-school children (PSC), Figure S2: The proportion of anemia associated with ID stratified by region among PSC, Figure S3: The proportion of anemia associated with ID stratified by rural/urban setting among PSC, Figure S4: The proportion of anemia associated with ID stratified by anemia public health significance among PSC, Figure S5: The proportion of anemia associated with ID stratified by inflammation exposure among PSC, Figure S6: Overall proportion of anemia associated with ID among women of reproductive age (WRA), Figure S7: The proportion of anemia associated with ID stratified by region among WRA, Figure S8: The proportion of anemia associated with ID stratified by rural/urban setting among WRA, Figure S9: The proportion of anemia associated with ID stratified by anemia public health significance among WRA, Figure S10: The proportion of anemia associated with ID stratified by inflammation exposure among WRA, Table S1: Inflammation exposure country categorization PSC and WRA.
