# Supplementary material for: The Proportion of Anemia Associated with Iron Deficiency in Low, Medium, and High Human Development Index Countries: A Systematic Analysis of National Surveys
Source: Nutrients. 2016 Nov 2;8(11):693. doi: 10.3390/nu8110693 (PMC5133080; doi:10.3390/nu8110693)
Supplement: Supplementary file 1 [file nutrients-08-00693-s001.zip › nutrients-150741 si for proofreading.pdf]

Supplementary Materials: The Proportion of Anemia Associated with Iron Deficiency in Low, Medium, and High Human Development Index Countries: A Systematic Analysis of National Surveys

Nicolai Petry, Ibironke Olofin, Richard F. Hurrell, Erick Boy, James P. Wirth, Mourad Moursi, Moira Donahue Angel and Fabian Rohner

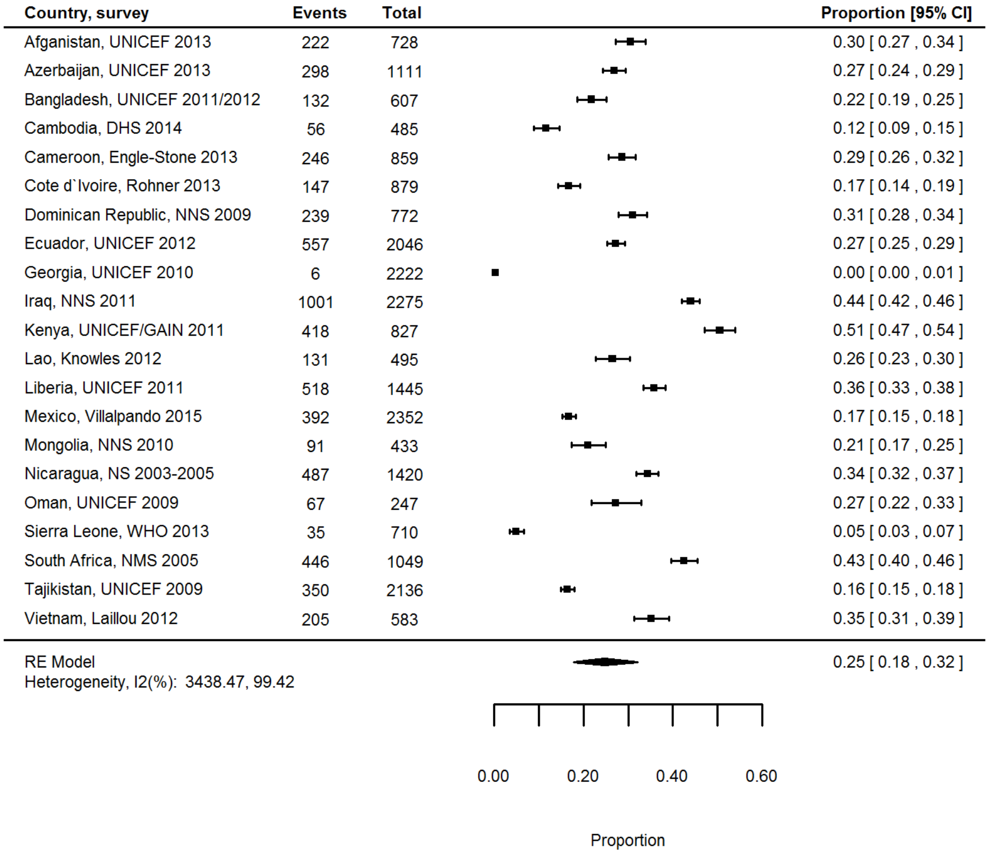

Figure S1. Overall proportion of anemia associated with iron deficiency (ID) among pre-school children (PSC).

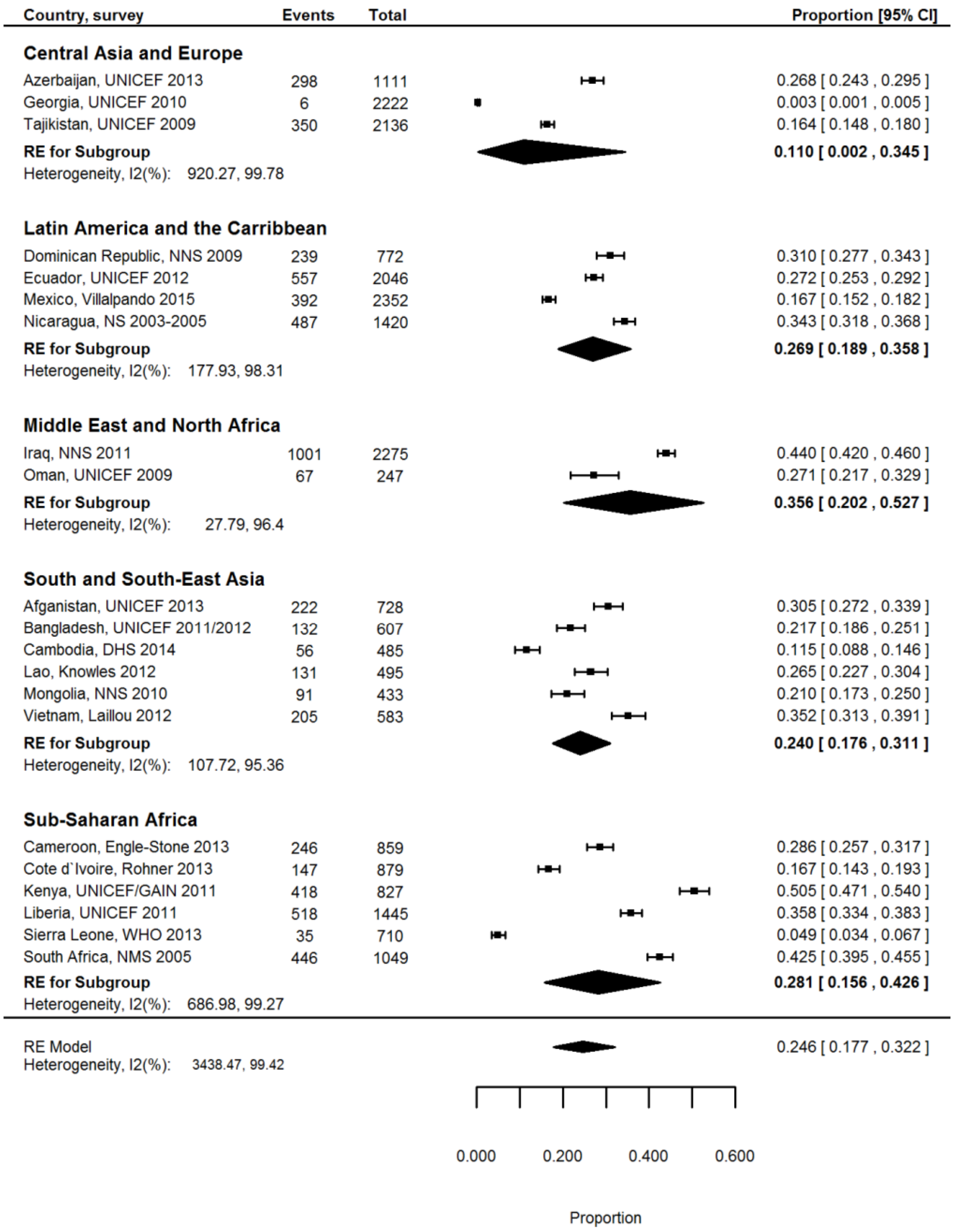

Figure S2. The proportion of anemia associated with ID stratified by region among PSC.

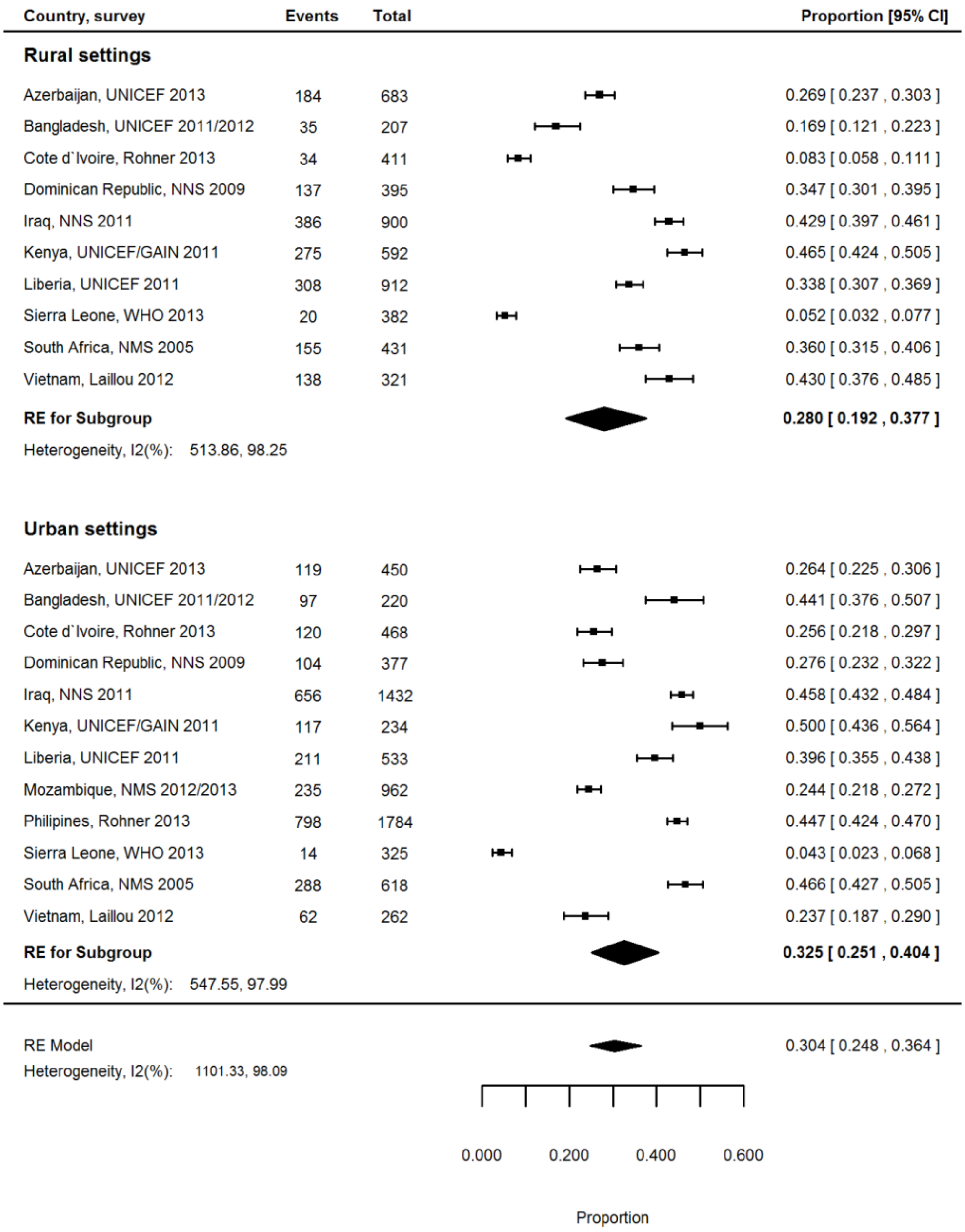

Figure S3. The proportion of anemia associated with ID stratified by rural/urban setting among PSC.

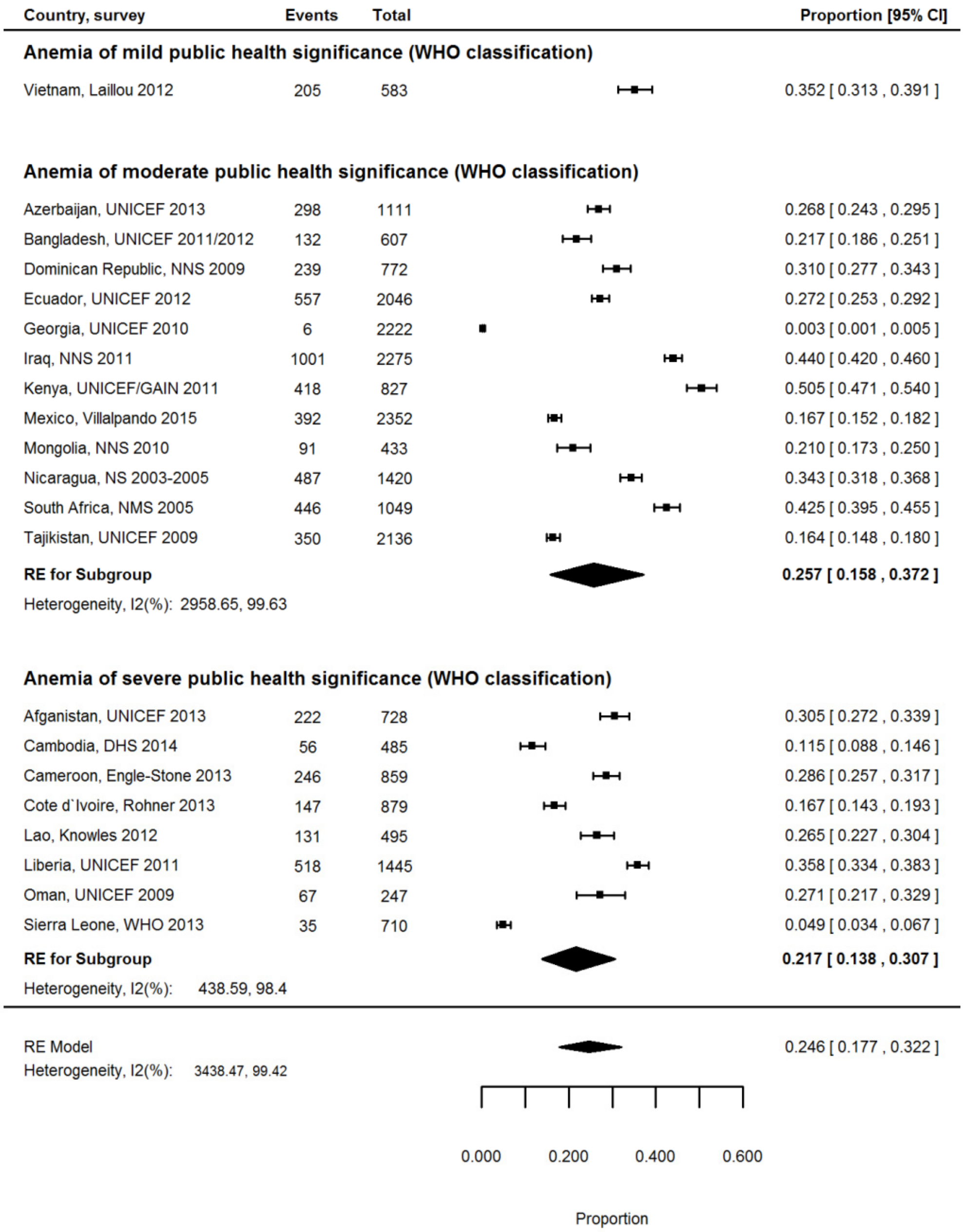

Figure S4. The proportion of anemia associated with ID stratified by anemia public health significance among PSC.

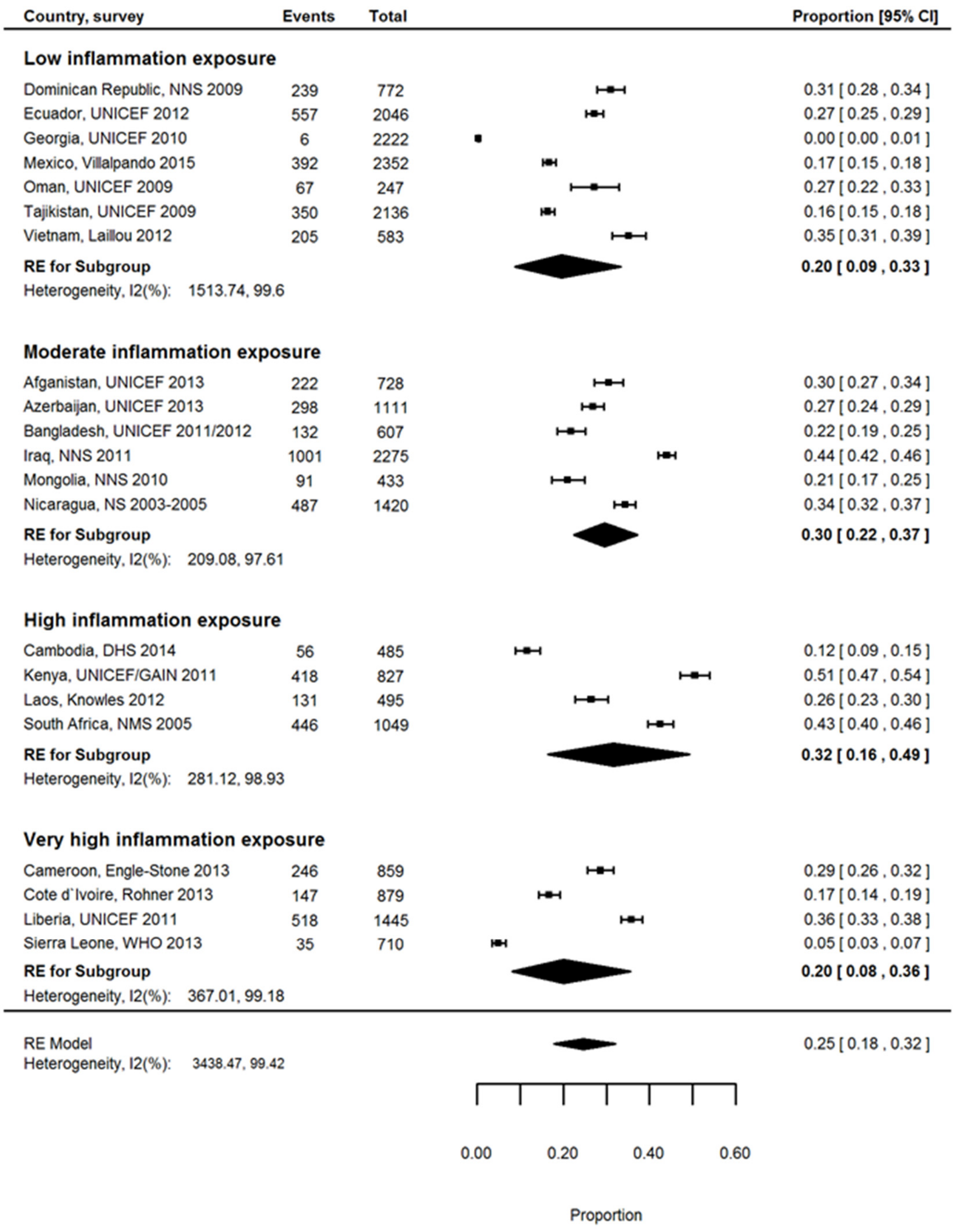

Figure S5. The proportion of anemia associated with ID stratified by inflammation exposure among PSC.

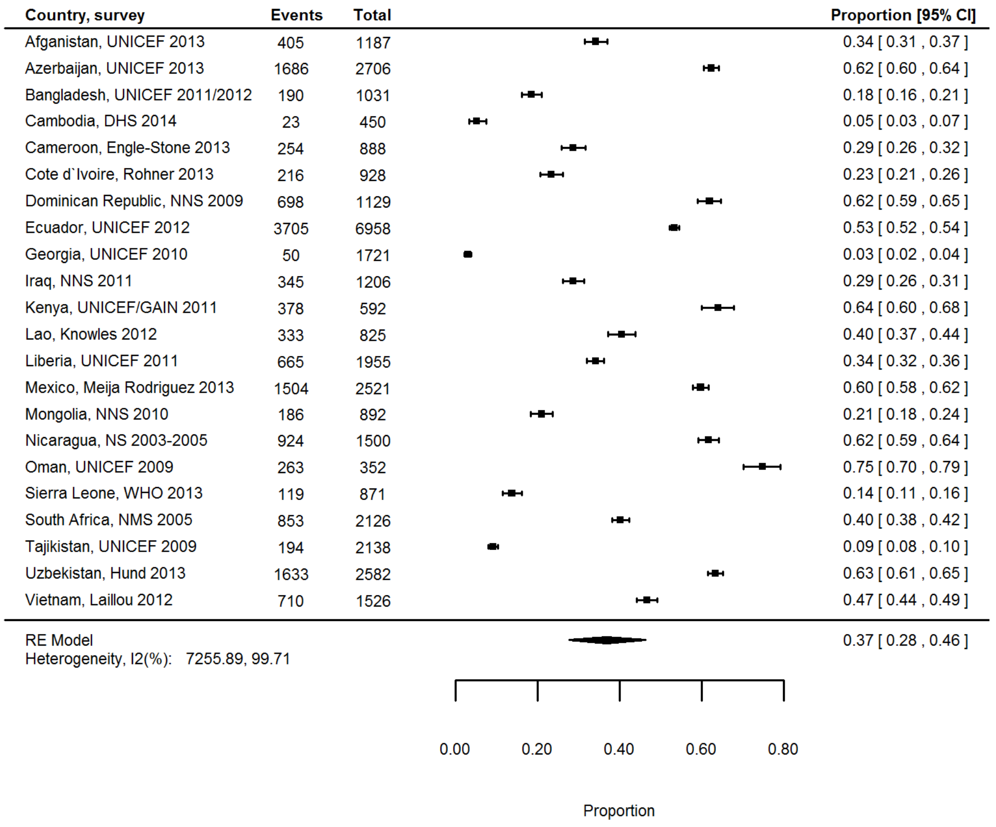

Figure S6. Overall proportion of anemia associated with ID among women of reproductive age (WRA).

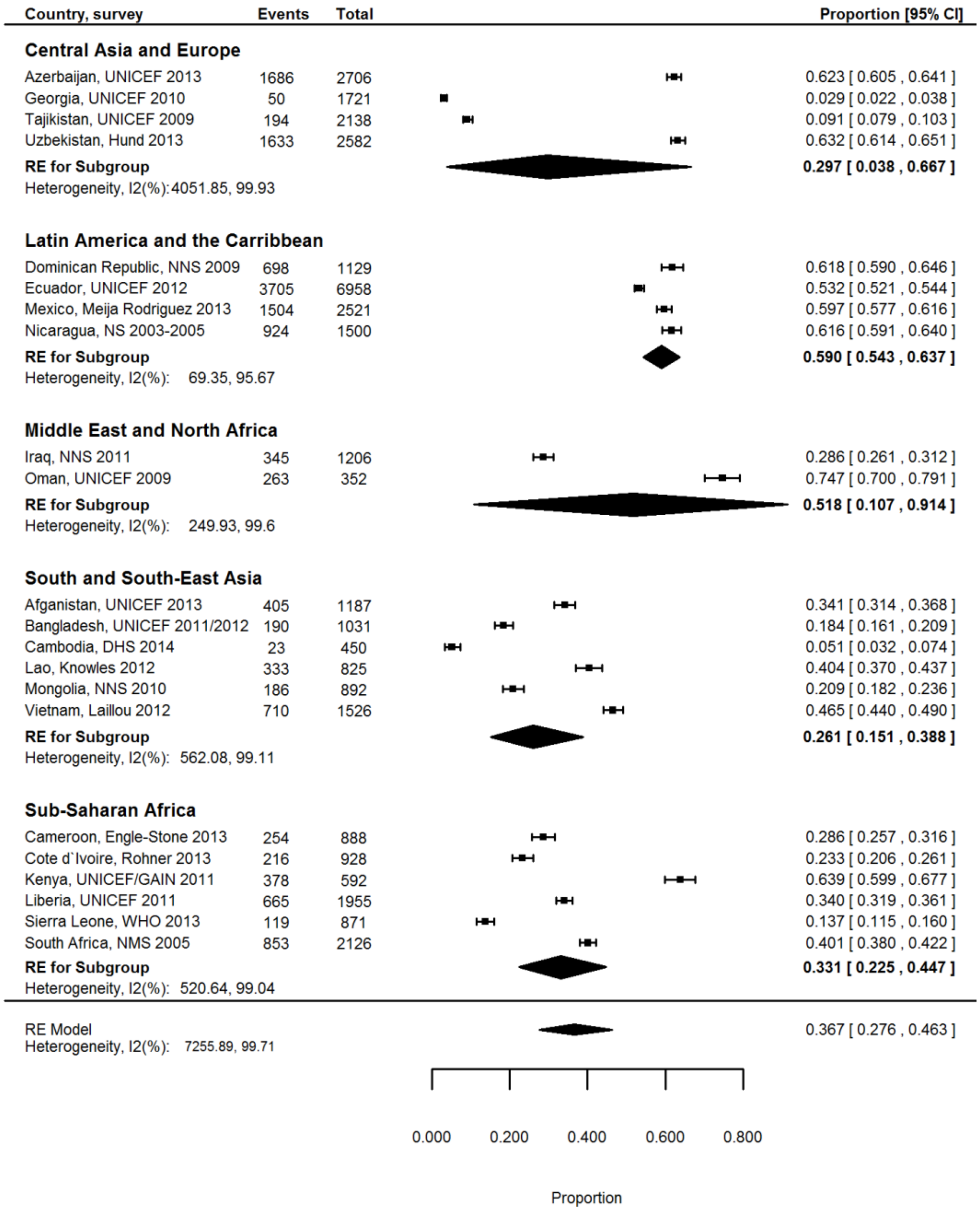

Figure S7. The proportion of anemia associated with ID stratified by region among WRA.

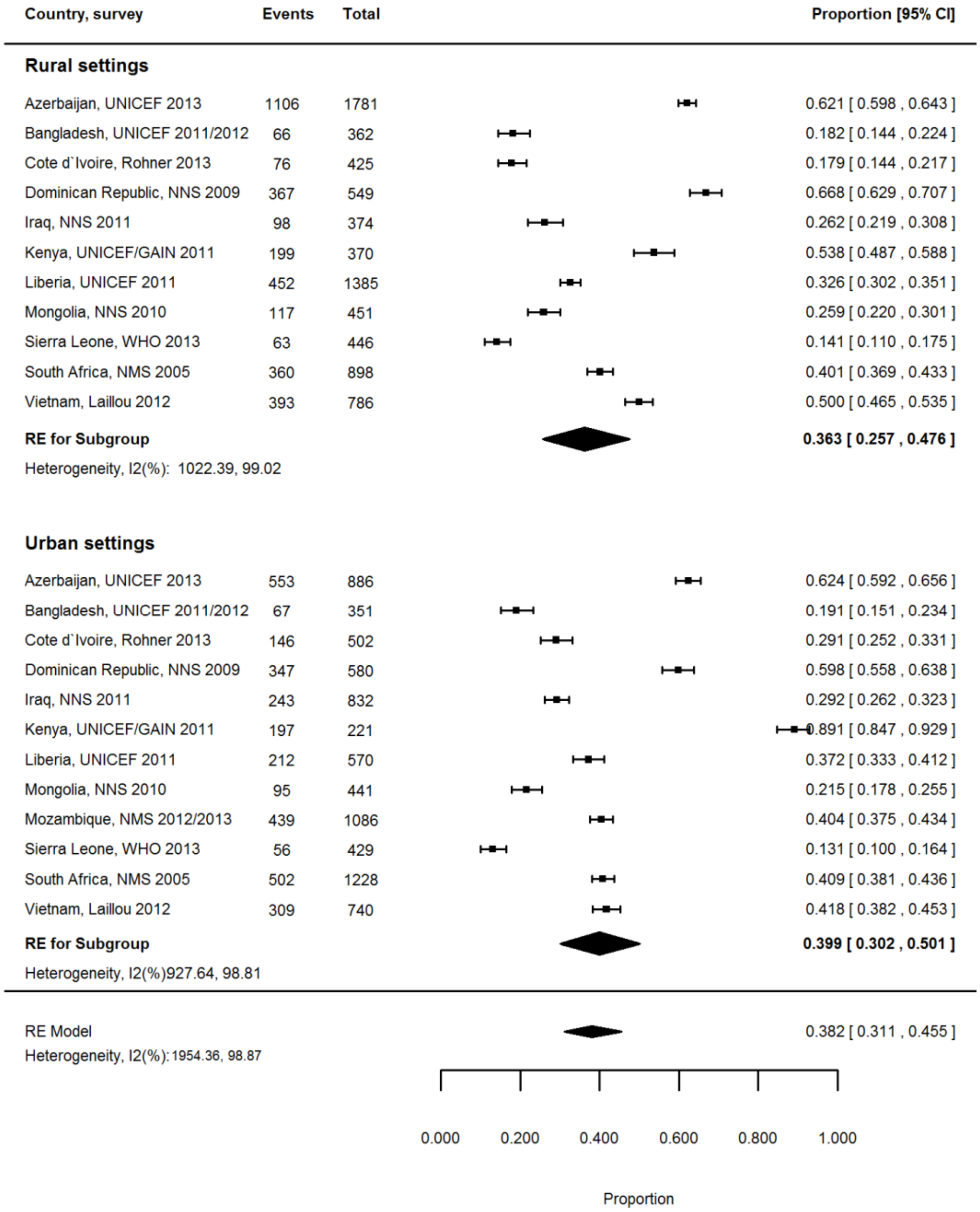

Figure S8. The proportion of anemia associated with ID stratified by rural/urban setting among WRA.

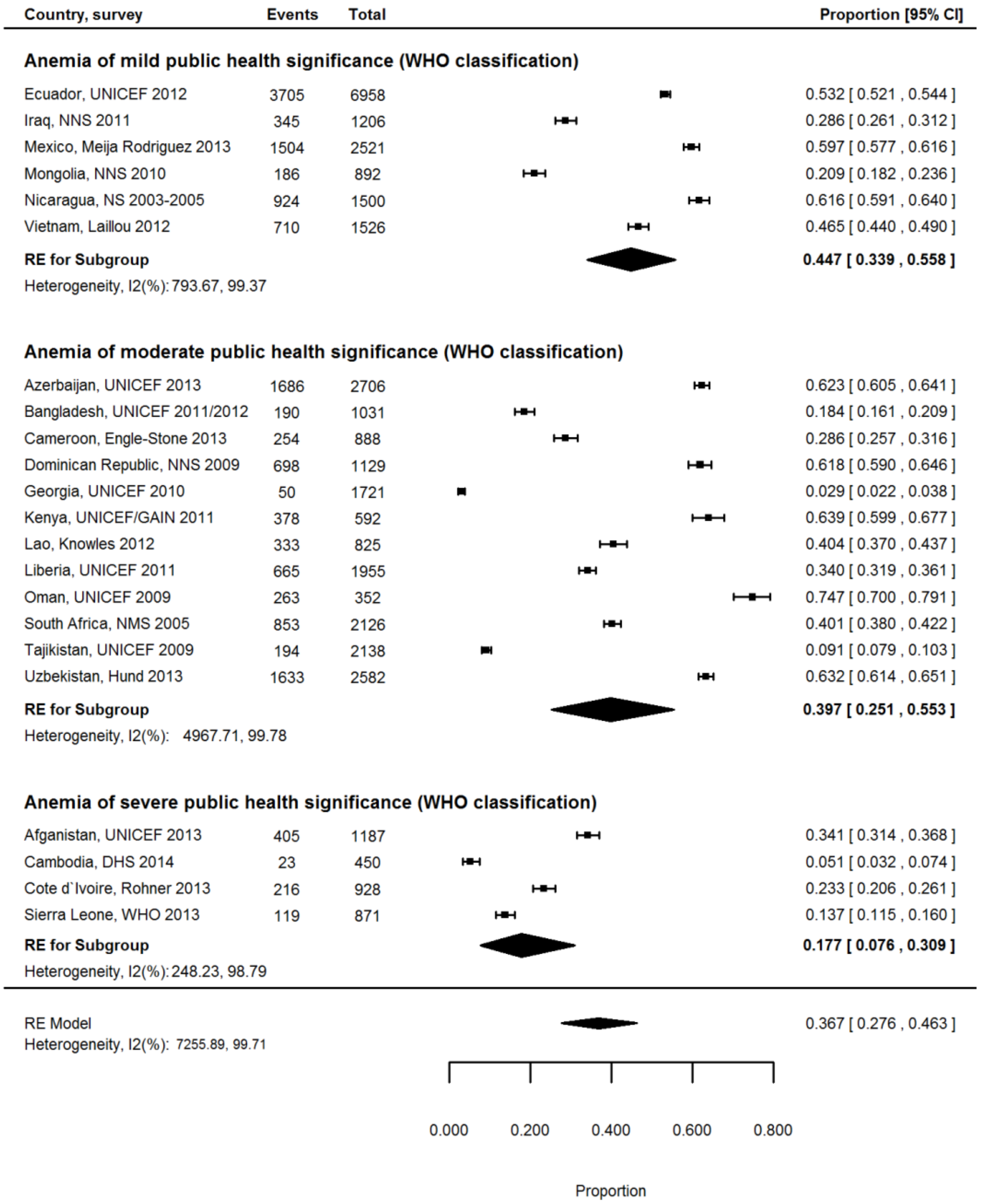

Figure S9. The proportion of anemia associated with ID stratified by anemia public health significance among WRA.

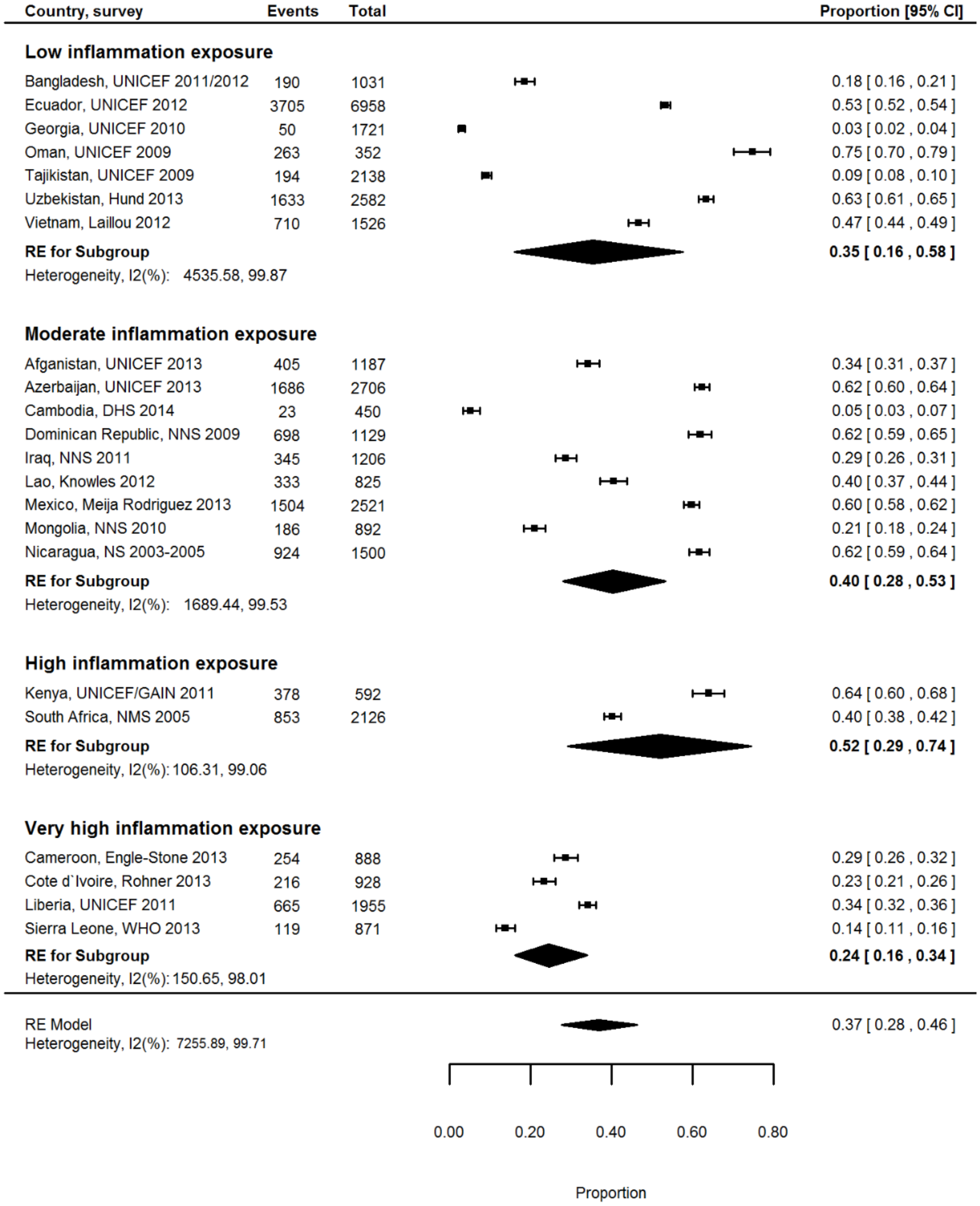

Figure S10. The proportion of anemia associated with ID stratified by inflammation exposure among WRA.
